# Endovascular recanalization for filter-bearing inferior vena cava occlusion in a dialysis patient

**DOI:** 10.1016/j.radcr.2024.08.007

**Published:** 2024-08-27

**Authors:** Ivana Boktor, Ahmed E. Ali, Ammar Almehmi

**Affiliations:** a George Walton Comprehensive High School, Marietta, GA, USA; b Internal Medicine Residency Program, Crestwood Medical Center, Huntsville, AL, USA; c Department of Medicine and Radiology, University of Alabama at Birmingham, Birmingham, AL, USA

**Keywords:** Vascular access, Dialysis, Central venous occlusion, Stenting

## Abstract

Central venous occlusion (CVO) or stenosis (CVS) is a common complication of long-term hemodialysis catheters. Endovascular intervention, primarily balloon angioplasty and occasionally stent placement, is the primary approach for managing CVS/CVO lesions. The presence of a filter within the inferior vena cava (IVC) lumen makes recanalization of the IVC more challenging. Here we present a complex case of a 47-year-old female with end-stage kidney disease (ESKD), systemic lupus erythematosus, and recurrent deep venous thrombosis, necessitating an IVC filter, who became catheter-dependent via the right femoral vein and presented with total IVC occlusion below the filter. The occlusion was managed successfully with sequential angioplasty and stenting of the stenotic lesions. This intervention restored venous flow through the IVC into the right atrium and maintained dialysis access through the catheter. This case underscores the complexity of managing CVS/CVO in dialysis patients, especially with the presence of filters within the vascular dialysis conduit.

## Introduction

Hemodialysis vascular access complications pose significant challenges in the management of end-stage kidney disease (ESKD) patients, particularly those with a history of multiple vascular comorbidities [1]. Effective vascular access is crucial for the survival and quality of life of hemodialysis patients, and when traditional options are exhausted, femoral vein catheters become necessary, albeit with associated risks [2]. Central venous occlusion (CVO) or stenosis (CVS) is a common complication of long-term hemodialysis catheters that can occur in the major veins such as the subclavian vein, superior vena cava (SVC), and inferior vena cava (IVC) leading to significant morbidity and mortality [3,4]. Further, these complications can preclude the creation of future vascular access [5]. Hence, catheter-sparing approaches in dialysis patients are highly recommended. Endovascular intervention is considered the primary approach to manage and treat CVS/CVO lesions, using mainly balloon angioplasty and occasionally stent placement [6]. On the other hand, the presence of a filter within the IVC lumen contributes to the thrombosis and makes establishing the venous flow more challenging. In order to address these lesions, the IVC filter must be dilated with a balloon, which carries the risk of rupturing the vessel. Therefore, recanalization of the IVC in the presence of a permanent filter poses several challenges, especially among those who exhaust other access options and other treatment modalities such as transplant and peritoneal dialysis. In this report, we present an unusual case with a complex medical history, including ESKD, systemic lupus erythematosus, and recurrent deep venous thrombosis (DVT) requiring an IVC filter, who became catheter-dependent via the right femoral vein and presented with total occlusion of IVC below the filter level.

## Case presentation

**Fig. 1:**
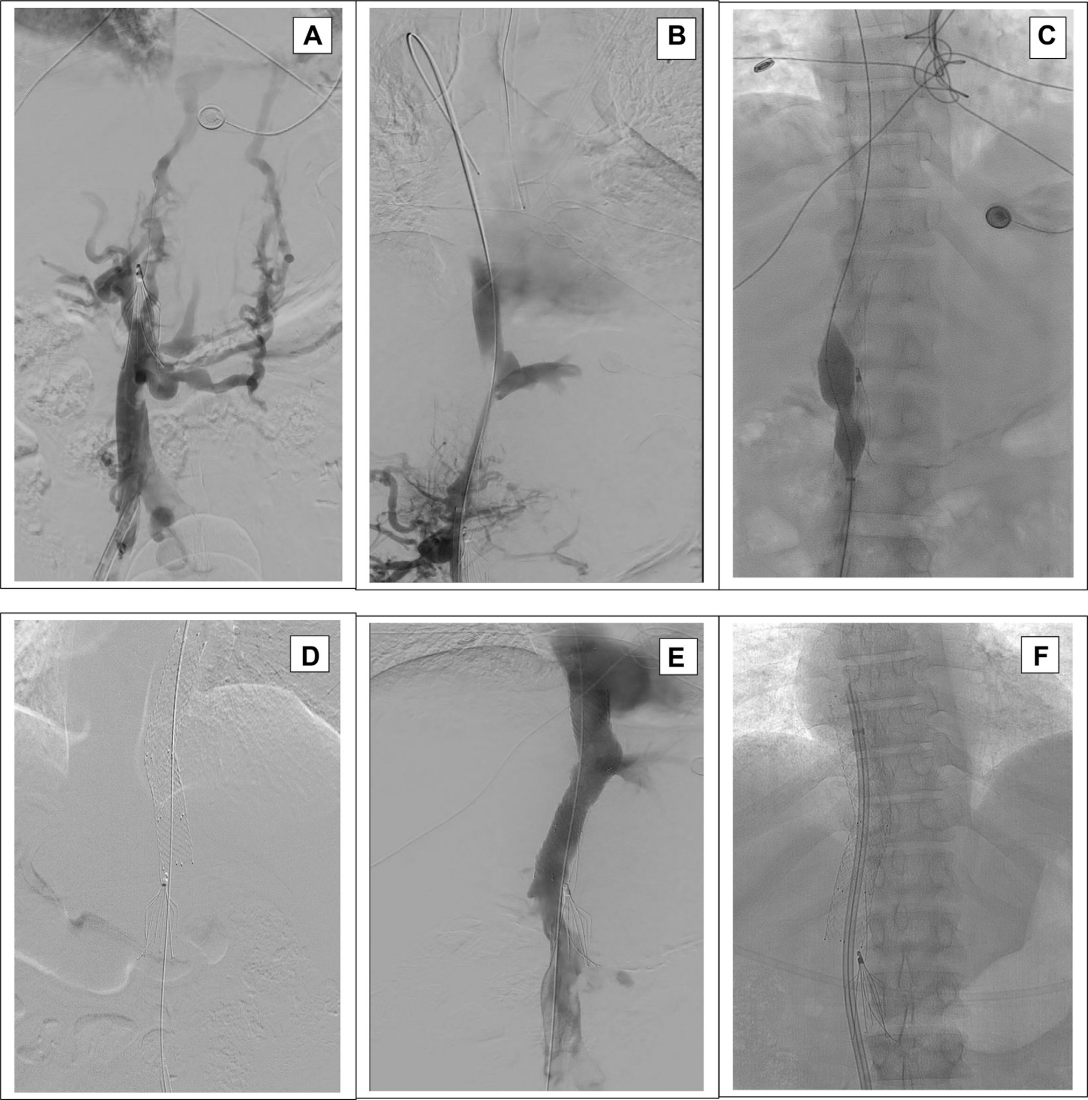
(A) venogram showing total occlusion of the suprarenal IVC vein above the filter; (B) recanalization of the IVC vein below the cavoatrial junction; (C) balloon angioplasty of the stenotic IVC lesions after stenting; (D) IVC stents above the filter level; (E) venogram showing patent IVC; (F) dialysis catheter tip is within the right atrium.

A 47-year-old African American female on chronic hemodialysis presented with a poorly functioning right femoral dialysis catheter. Past medical history was remarkable for ESKD in 1996 due to pre-eclampsia, systemic lupus erythematosus complicated with multiple DVT of the lower extremities, treated with IVC filter placement and lifelong chronic oral anticoagulation, and status post kidney transplant between 2009 and 2015, during which she was off dialysis. Her medications included aspirin, Apixaban, pantoprazole, and calcitriol, and she was reported to be adherent to her anticoagulation therapy per her managing nephrologist. After failing peritoneal dialysis and multiple dialysis accesses in both upper and lower extremities, she became catheter-dependent via the right femoral vein. Over the last 5 years, she underwent more than 50 catheter exchanges. The patient was referred to our center after failing attempts to remove the IVC filter and recanalizing the occlusion by another interventional group despite using both right internal jugular and femoral approaches. During the current presentation, the venogram through the existing catheter demonstrated complete occlusion of the IVC and extensive collateralization (Fig. 1A). After multiple attempts of using a Kumpe catheter and different wires, the supra-renal IVC was recanalized, and the wire was negotiated under fluoroscopy guidance into the right atrium (Fig. 1B). After sequential angioplasty of the stenotic lesions (Fig. 1C), a 16 × 100 mm Zilver stent (Cook Medical LLC, Bloomington, IN) was deployed from the inferior cavoatrial junction to the mid SVC. This was followed by a second overlapping stent deployment (16×60mm Zilver) along the inferior margin of the first stent and extending to the superior margin of the IVC filter (Fig. 1D). The final poststenting angiogram demonstrated patency through the stent with brisk blood flow into the right atrium, and no collateral flow was noted (Fig. 1E). Dialysis therapy was resumed immediately after the intervention (Fig. 1F), and the catheter has remained functioning up till now.

## Discussion

CVS/CVO is a serious complication that is frequently associated with long-term dialysis access use of CVCs [3,7]. Femoral vein catheters are considered one of the last options to provide dialysis therapy due to the increased risk of infection and other complications [8]. These catheters are associated with lower patency rates, increased infection rates, stenotic lesions, and occlusions of the IVC. Further, the presence of medical devices, such as IVC filters, within the dialysis conduit poses more challenges in managing these occlusions. Currently, the endovascular approach is the standard of care that is used to treat IVC occlusions and thrombosis such as mechanical thrombectomy, catheter-directed thrombolysis, and balloon venoplasty with stent placement [6].

In our case, the other dialysis access options were exhausted in both upper and lower extremities due to recurrent thrombosis; further, the peritoneal dialysis option was also excluded due to frequent infections. Due to the recurrent lower extremity thrombosis, an IVC filter was placed. In our patient, the initial position of the femoral catheter was under the filter level. However, the patient developed IVC thrombosis below the filter level leading to poor access flow despite being treated with oral anticoagulation, a complication that required moving the femoral catheter above the filter level. Expectedly, the new catheter position was complicated with stenosis and occlusion. Traditionally, these lesions are treated with balloon angioplasty. However, the presence of an IVC filter and the total occlusion of the IVC vein proximal to the filter necessitated stenting the IVC to secure the patency of the dialysis access. As such, the IVC conduit was re-canalized and subsequently dilated and stented to secure the patency of the vein above the filter level. Moreover, during this admission, the medical team considered removing the IVC filter; however, after consulting with the hematology team, the decision was made to retain the filter due to her high risk for pulmonary embolism. Alternative treatment modalities such as catheter-directed thrombolysis and surgical options were evaluated. Catheter-directed thrombolysis carries risks of bleeding and infection. Whereas, the surgical option is usually considered the last resort for those who failed endovascular treatment, had refractory clinical picture, or younger patients with lower comorbidities; hence, this option was deemed high-risk given the patient's comorbidities [5]. Sequential angioplasty and stenting provided a minimally invasive option with a more favorable risk-benefit profile. In this regard, it's noteworthy that a previous study comparing surgical bypass and percutaneous angioplasty with stent placement reported similar outcomes and mortality rates at a 1-year follow-up period [9].

Despite the patient has maintained a functioning dialysis catheter with no signs of recurrent occlusion or filter-related complications, it is expected that intra-stent stenosis and subsequent thrombosis are likely to develop, as with any vascular stent. So, regular monitoring of the dialysis access and anticoagulation therapy are essential for favorable long-term outcomes. In conclusion, our case highlights the challenges encountered in managing CVS/CVO lesions in dialysis patients who exhausted access options. The presence of an IVC filter within the vascular dialysis conduit adds another layer of complexity to managing the dialysis access dysfunction in this population. For dialysis patients, avoiding CVC is the best approach if feasible. In the presence of an IVC filter, perhaps removing the device, followed by angioplasty and stenting -if indicated- would be the preferred approach. Comparative studies on different treatment modalities could provide valuable insights into best practices, especially as the guidelines to manage CVS lesions, specifically in the presence of filters, are still lacking.

## Patient consent

Written informed consent was obtained from the patient for all procedures and publication of this case and accompanying images.
